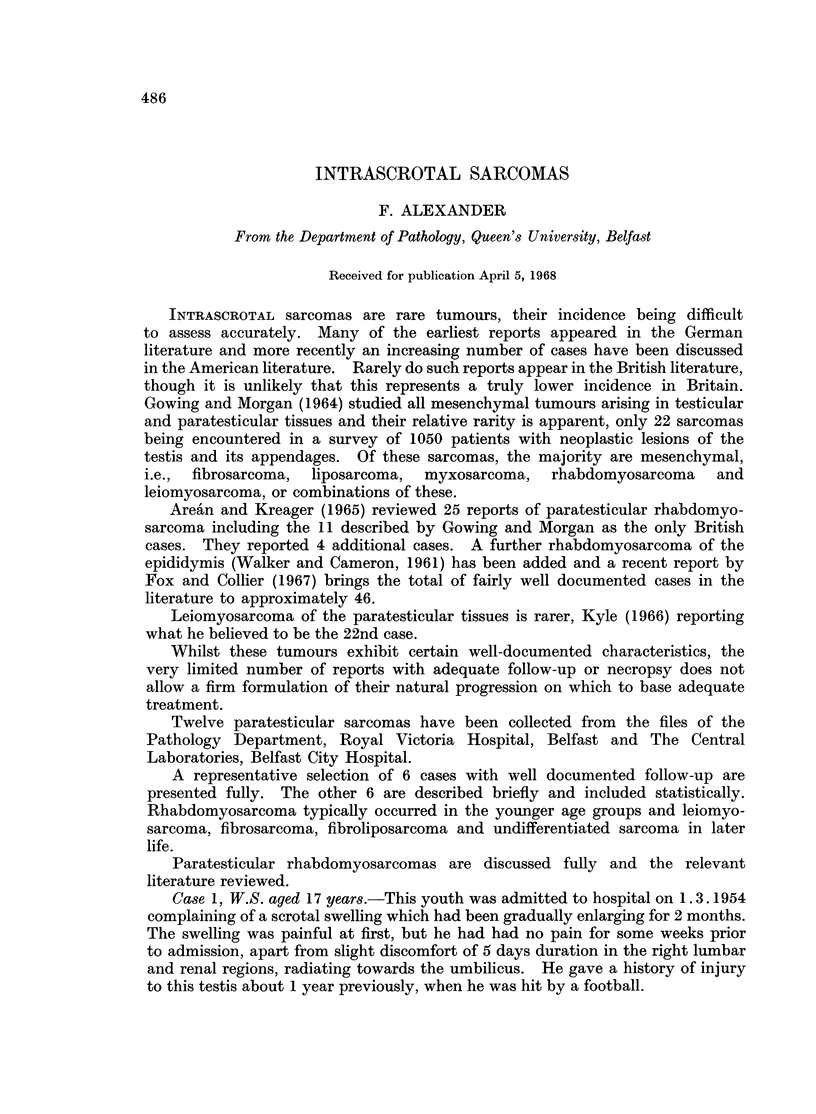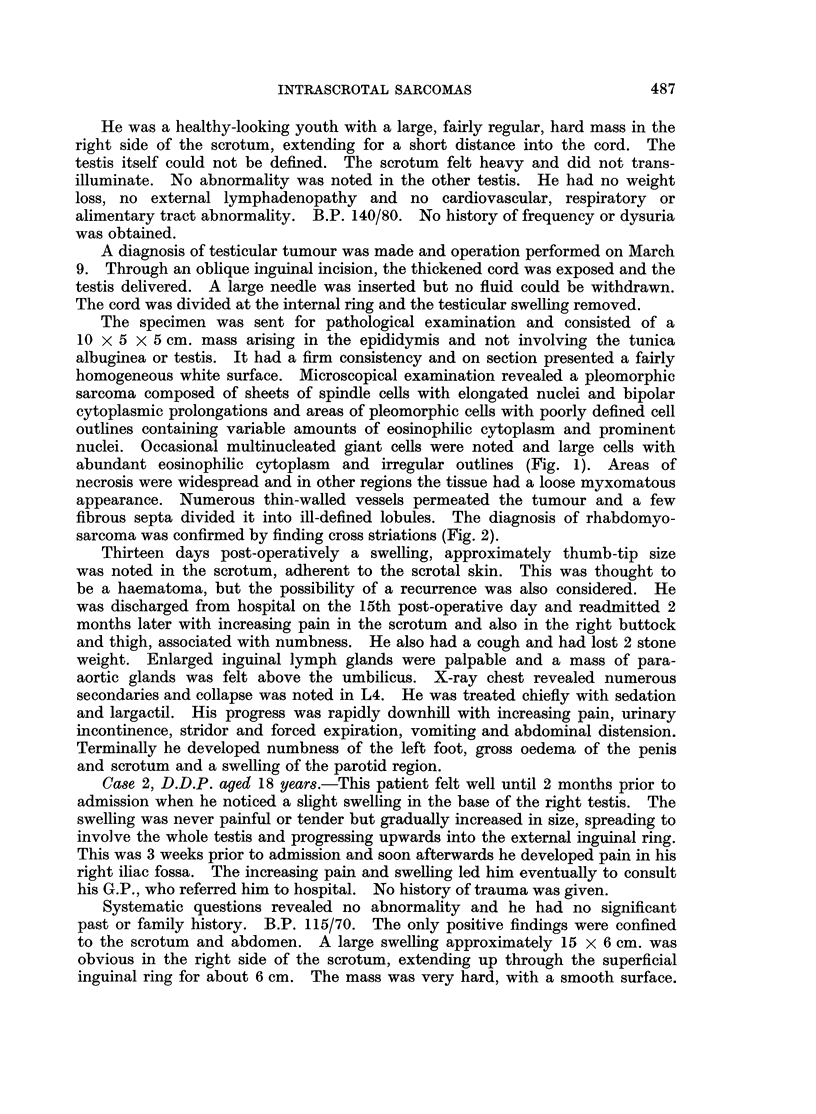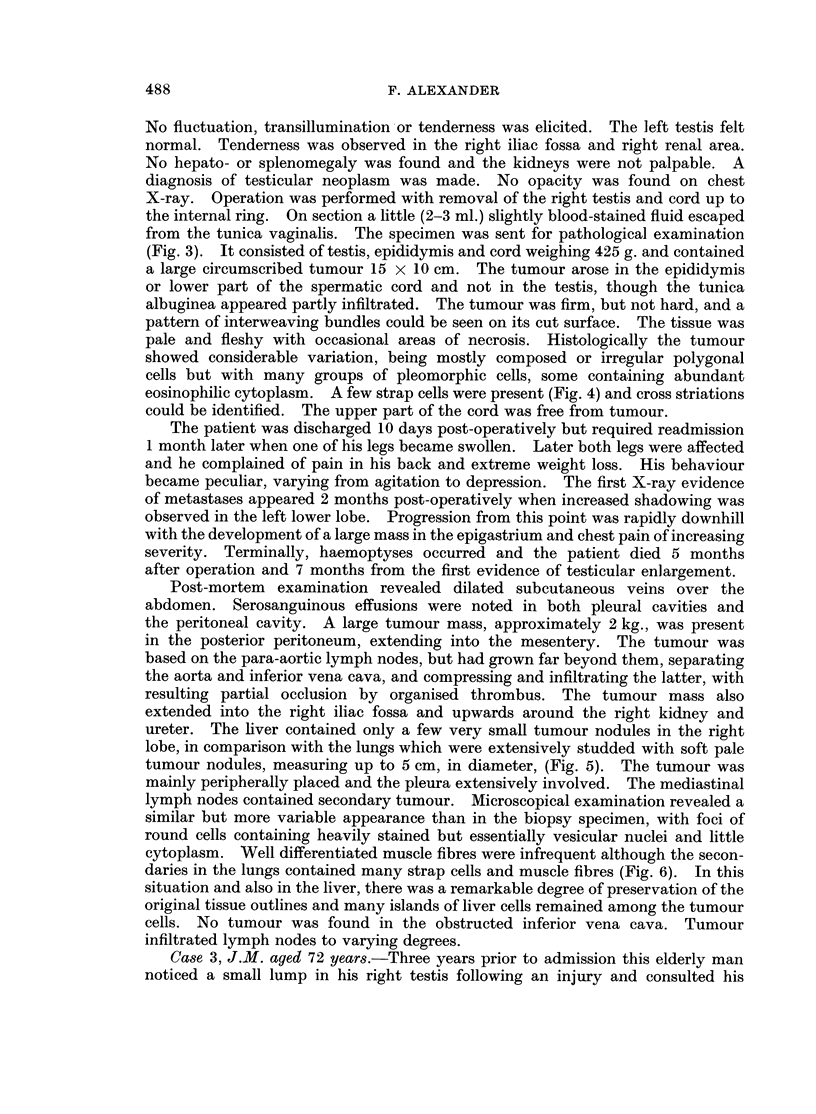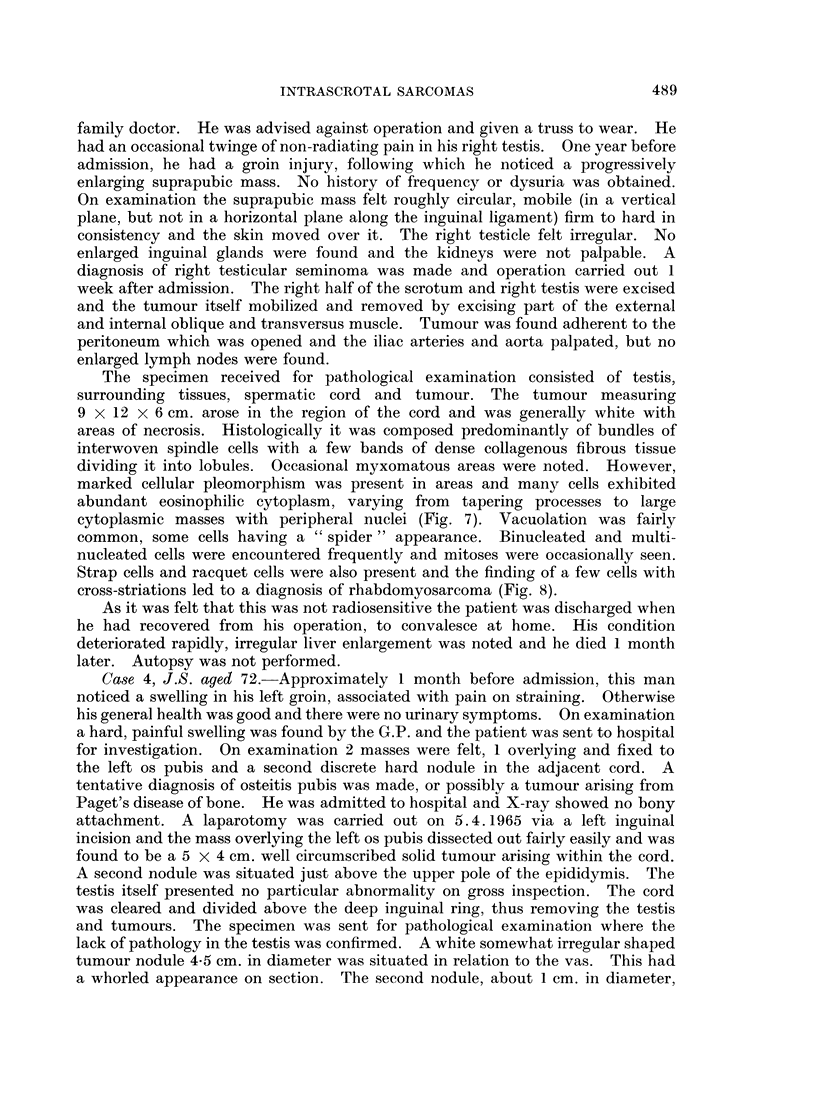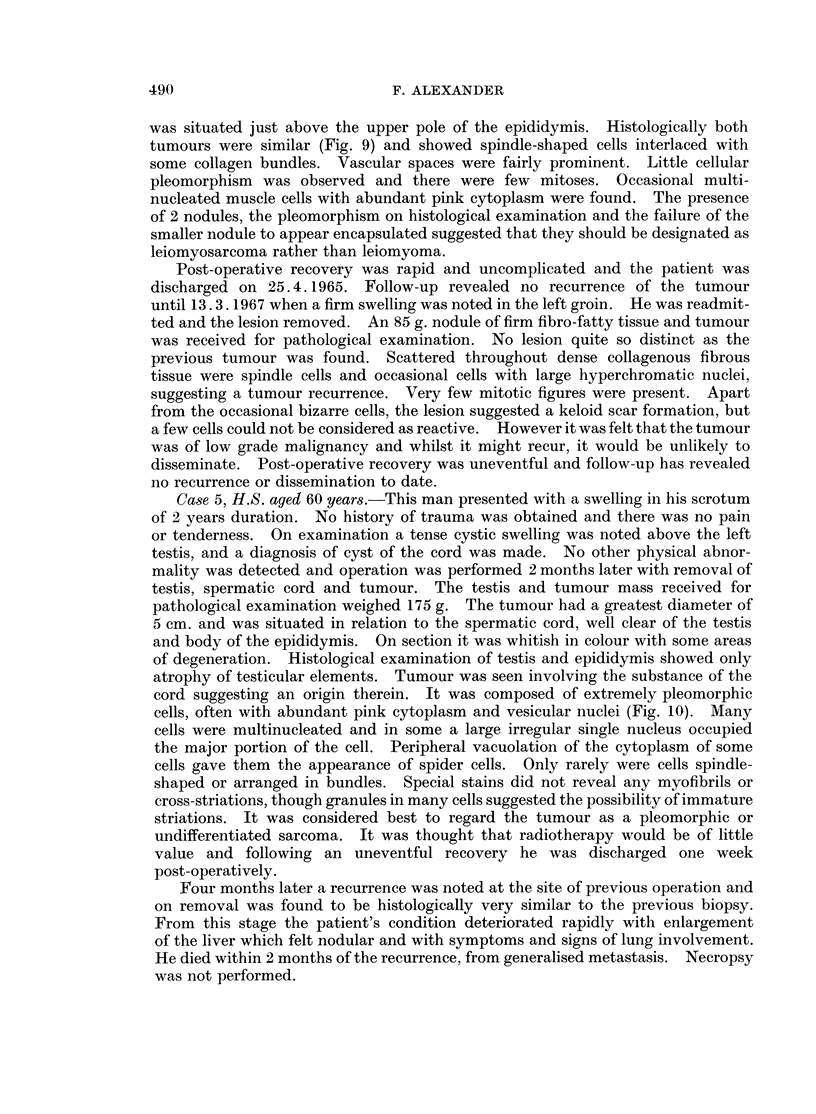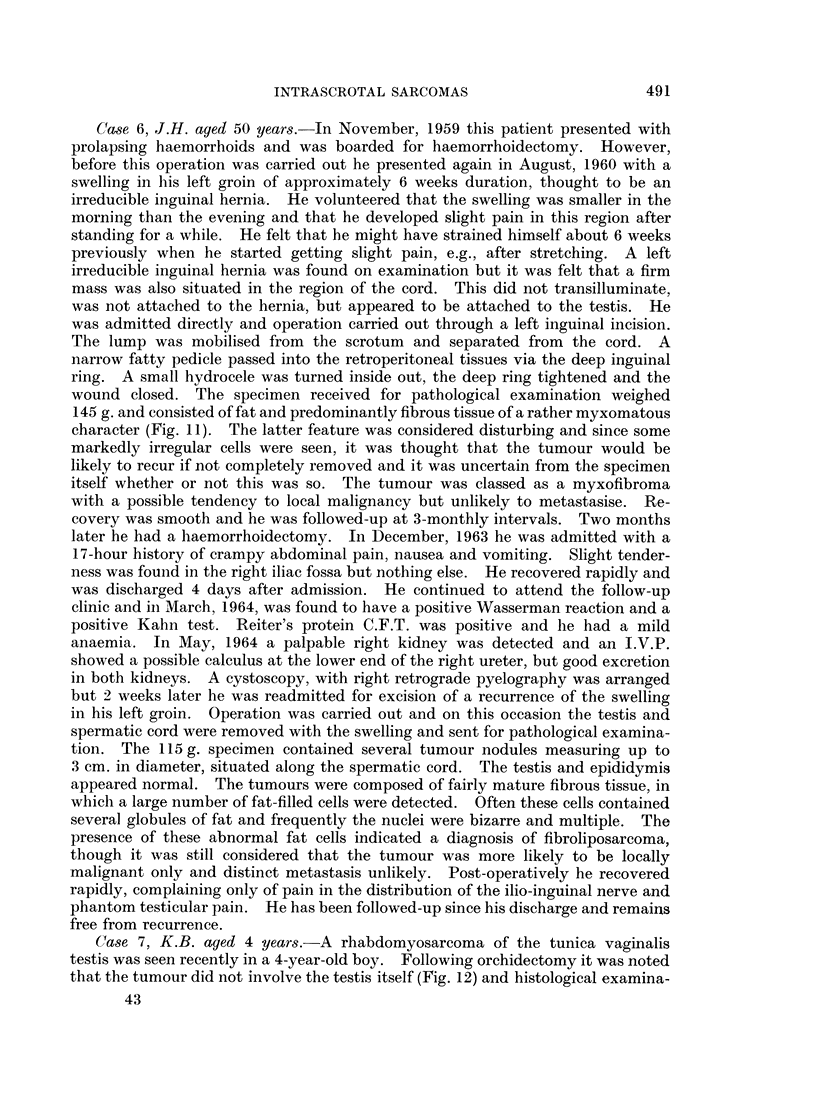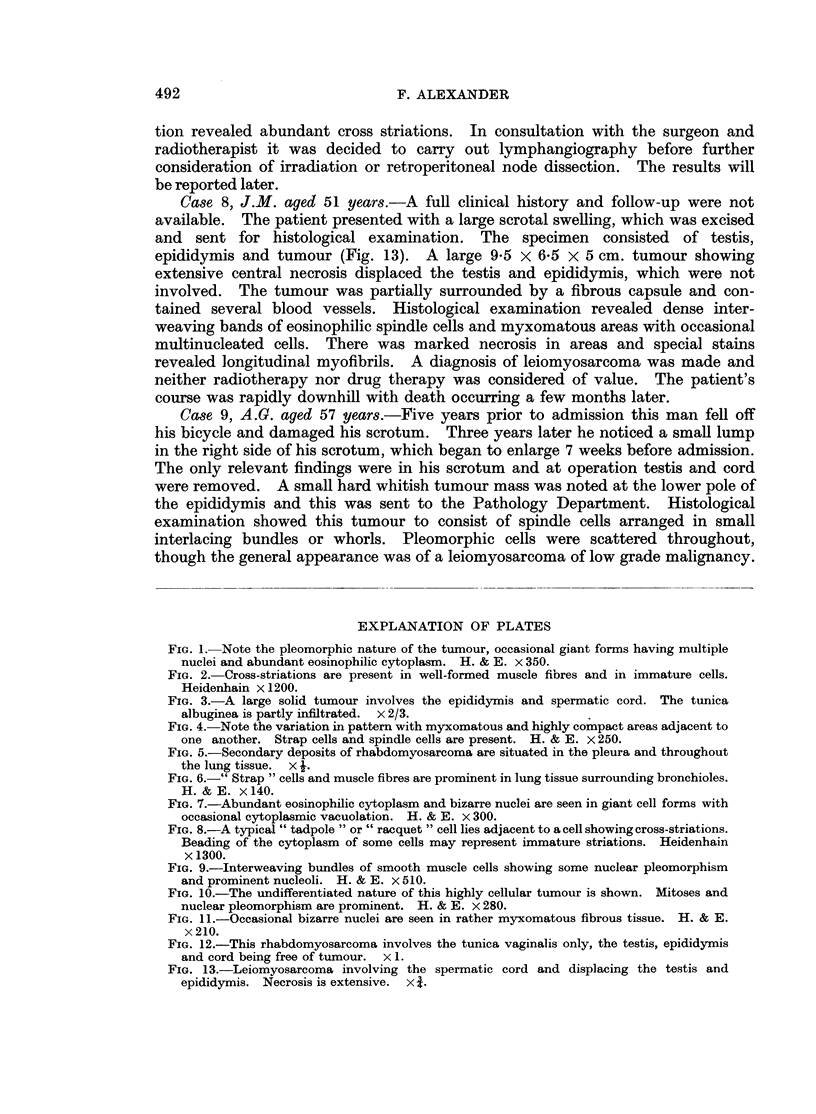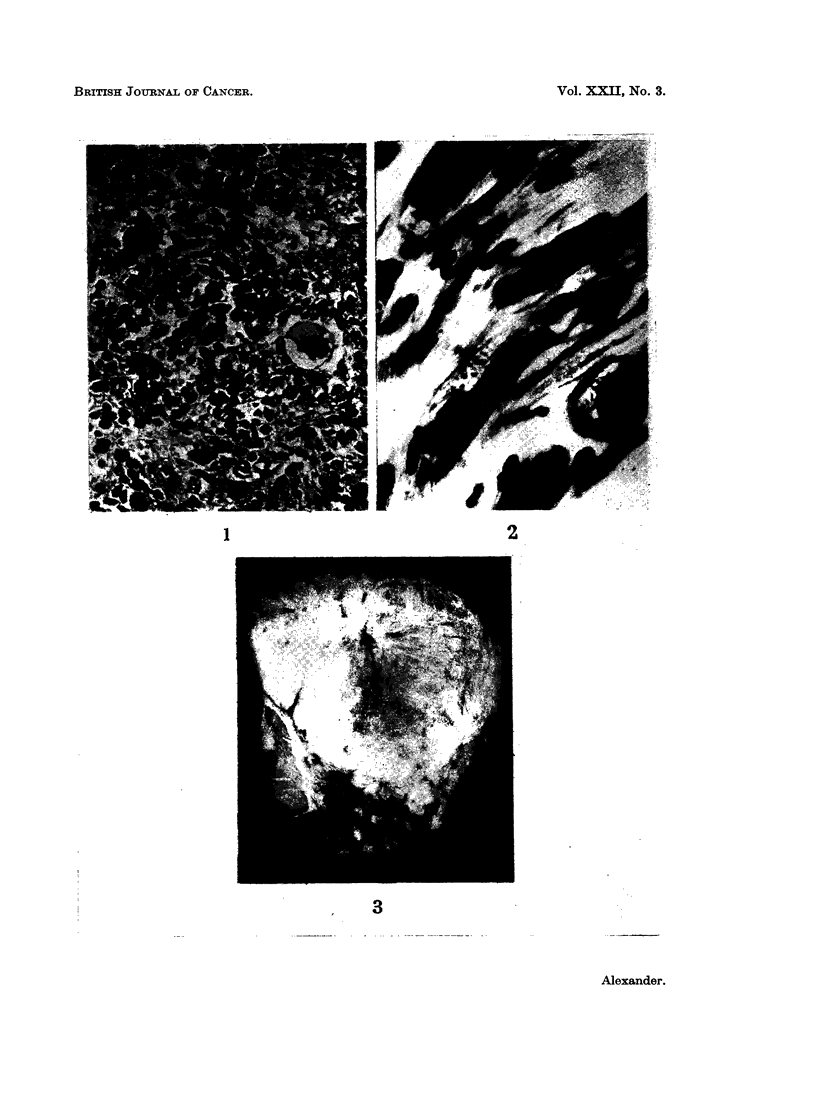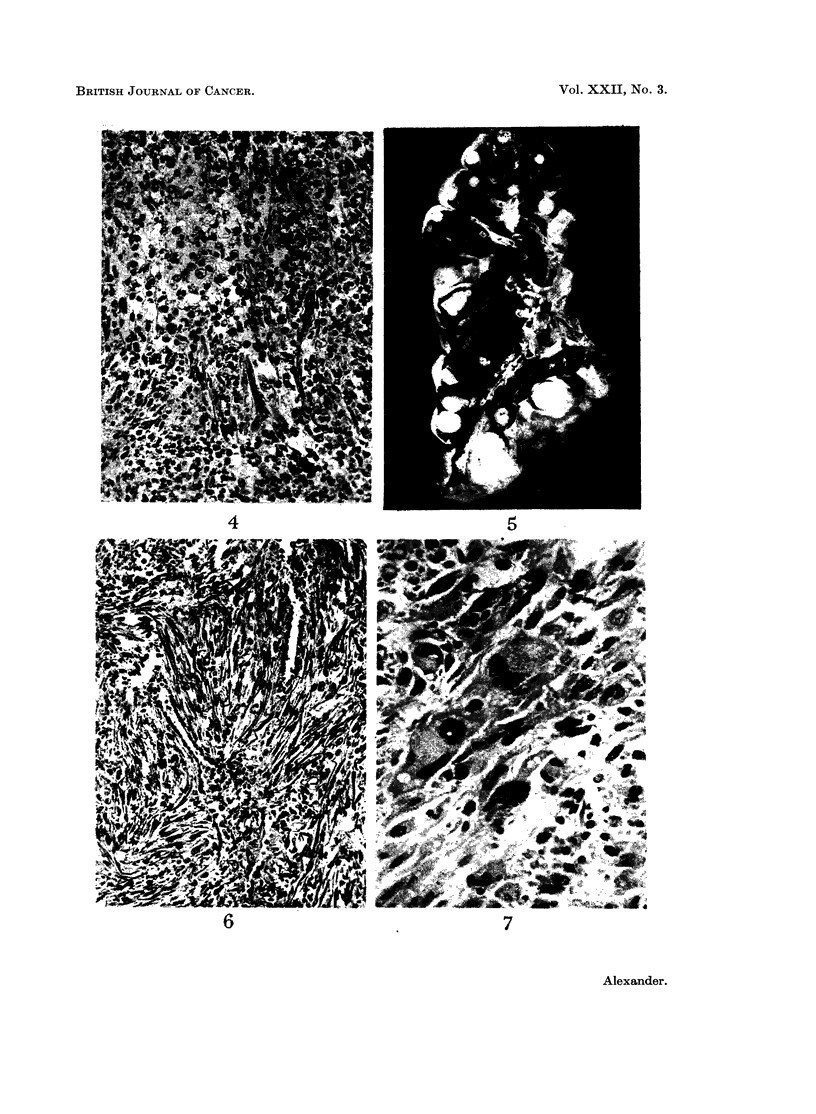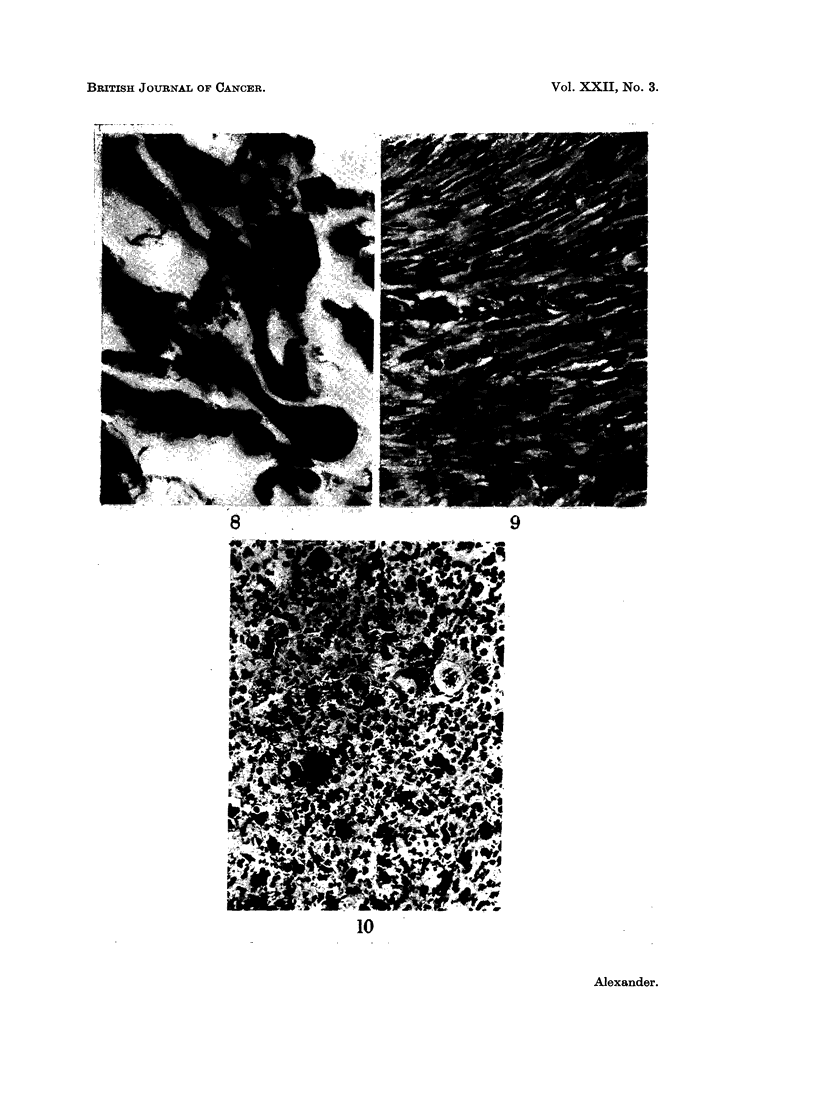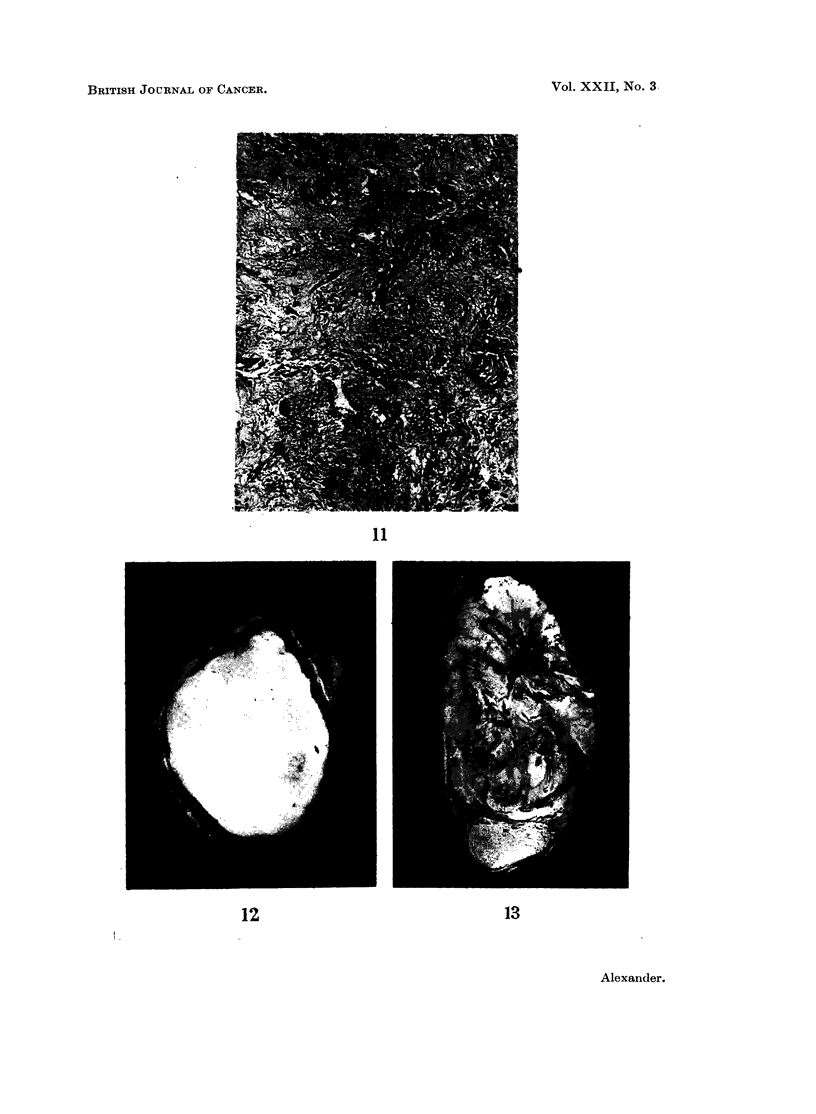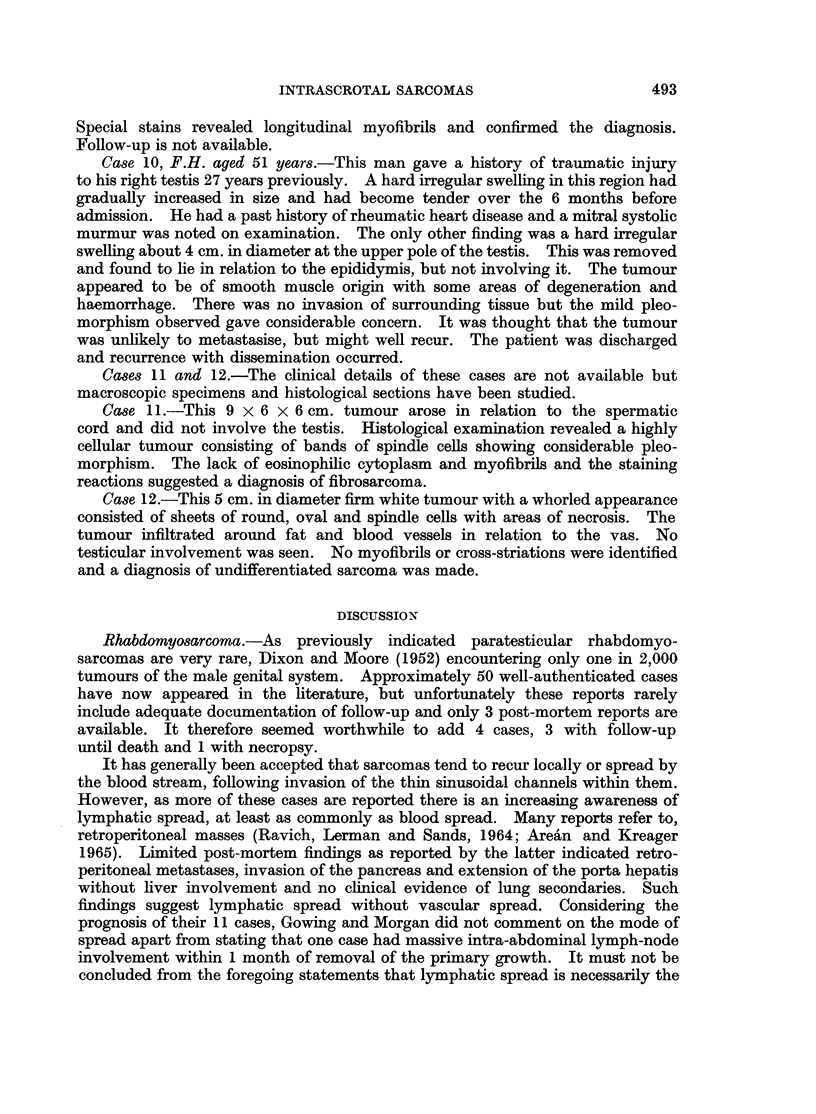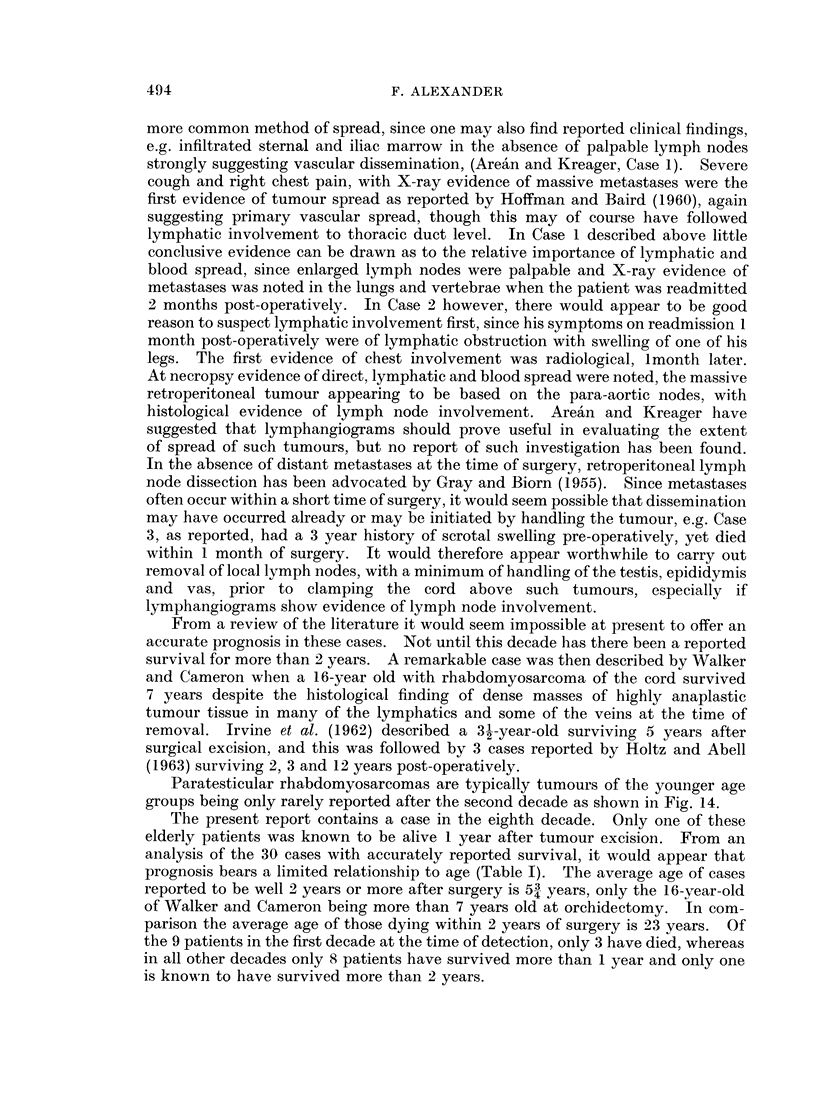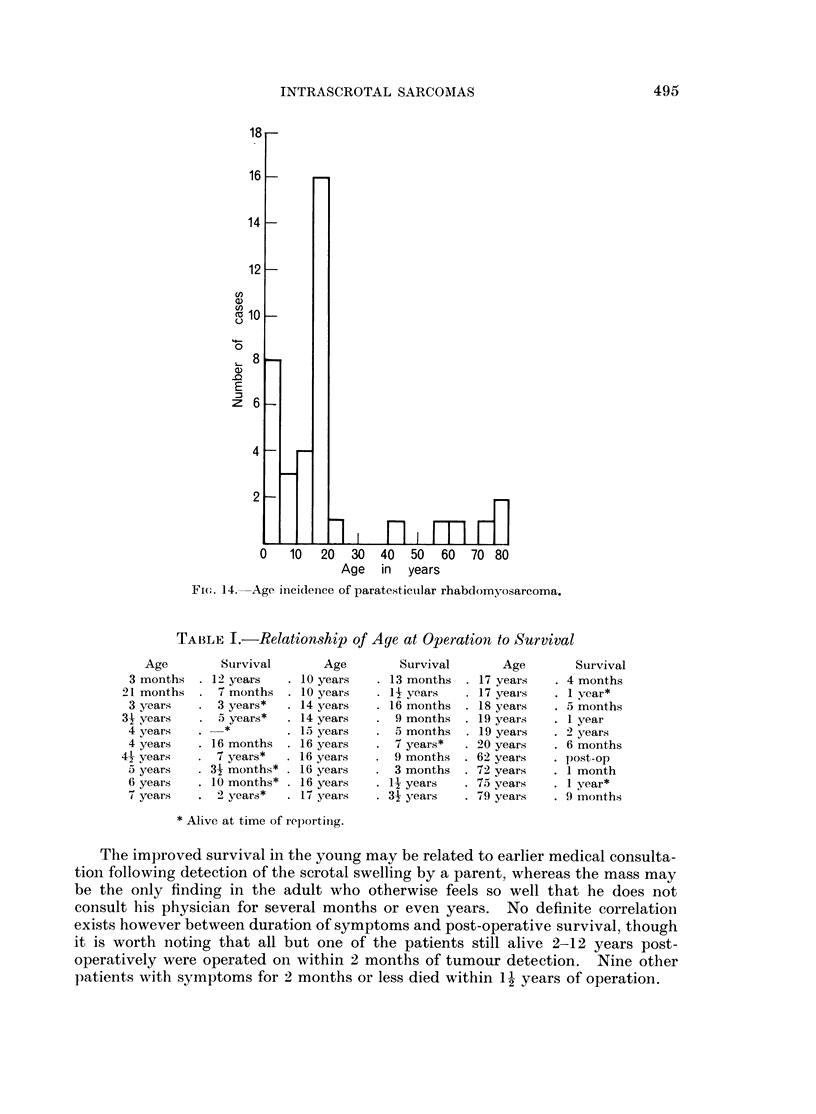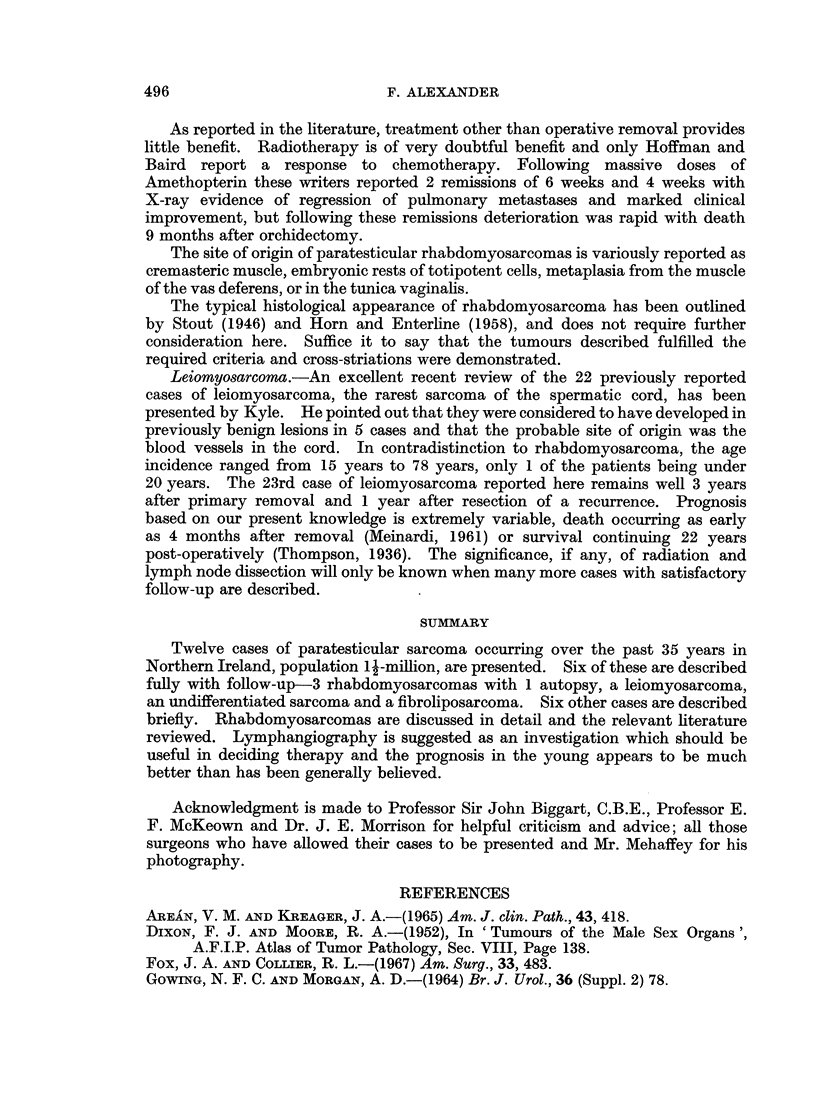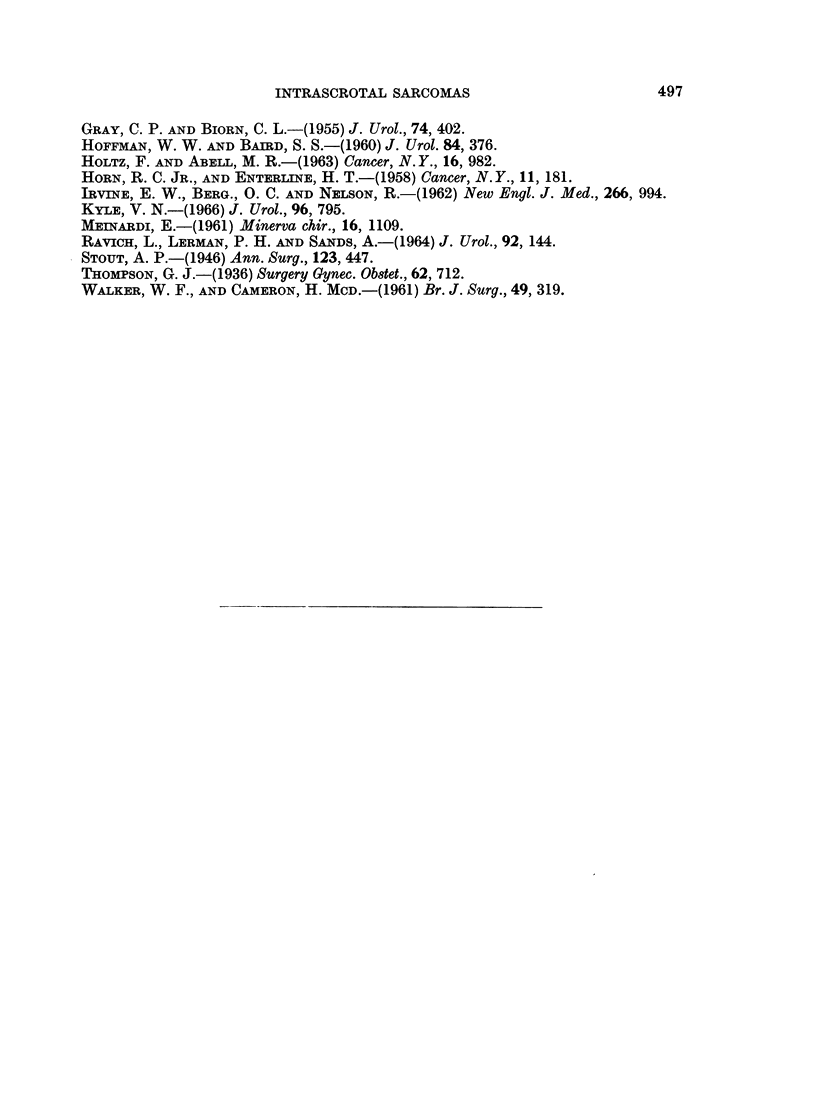# Intrascrotal sarcomas.

**DOI:** 10.1038/bjc.1968.57

**Published:** 1968-09

**Authors:** F. Alexander

## Abstract

**Images:**


### INTRASCROTAL SARCOMAS

F. ALEXANDER

*From the Department of Pathology, Queen's University, Belfast*

Received for publication April 5, 1968

INTRASCROTAL sarcomas are rare tumours, their incidence being difficult to assess accurately. Many of the earliest reports appeared in the German literature and more recently an increasing number of cases have been discussed in the American literature. Rarely do such reports appear in the British literature, though it is unlikely that this represents a truly lower incidence in Britain. Gowing and Morgan (1964) studied all mesenchymal tumours arising in testicular and paratesticular tissues and their relative rarity is apparent, only 22 sarcomas being encountered in a survey of 1050 patients with neoplastic lesions of the testis and its appendages. Of these sarcomas, the majority are mesenchymal, i.e., fibrosarcoma, liposarcoma, myxosarcoma, rhabdomyosarcoma and leiomyosarcoma, or combinations of these.

Areán and Kreager (1965) reviewed 25 reports of paratesticular rhabdomyosarcoma including the 11 described by Gowing and Morgan as the only British cases. They reported 4 additional cases. A further rhabdomyosarcoma of the epididymis (Walker and Cameron, 1961) has been added and a recent report by Fox and Collier (1967) brings the total of fairly well documented cases in the literature to approximately 46.

Leiomyosarcoma of the paratesticular tissues is rarer, Kyle (1966) reporting what he believed to be the 22nd case.

Whilst these tumours exhibit certain well-documented characteristics, the very limited number of reports with adequate follow-up or necropsy does not allow a firm formulation of their natural progression on which to base adequate treatment.

Twelve paratesticular sarcomas have been collected from the files of the Pathology Department, Royal Victoria Hospital, Belfast and The Central Laboratories, Belfast City Hospital.

A representative selection of 6 cases with well documented follow-up are presented fully. The other 6 are described briefly and included statistically. Rhabdomyosarcoma typically occurred in the younger age groups and leiomyosarcoma, fibrosarcoma, fibroliposarcoma and undifferentiated sarcoma in later life.

Paratesticular rhabdomyosarcomas are discussed fully and the relevant literature reviewed.

*Case* 1, *W.S. aged* 17 *years*.—This youth was admitted to hospital on 1.3.1954 complaining of a scrotal swelling which had been gradually enlarging for 2 months. The swelling was painful at first, but he had had no pain for some weeks prior to admission, apart from slight discomfort of 5 days duration in the right lumbar and renal regions, radiating towards the umbilicus. He gave a history of injury to this testis about 1 year previously, when he was hit by a football.

He was a healthy-looking youth with a large, fairly regular, hard mass in the right side of the scrotum, extending for a short distance into the cord. The testis itself could not be defined. The scrotum felt heavy and did not transilluminate. No abnormality was noted in the other testis. He had no weight loss, no external lymphadenopathy and no cardiovascular, respiratory or alimentary tract abnormality. B.P. 140/80. No history of frequency or dysuria was obtained.

A diagnosis of testicular tumour was made and operation performed on March 9. Through an oblique inguinal incision, the thickened cord was exposed and the testis delivered. A large needle was inserted but no fluid could be withdrawn. The cord was divided at the internal ring and the testicular swelling removed.

The specimen was sent for pathological examination and consisted of a $10 \times 5 \times 5$ cm. mass arising in the epididymis and not involving the tunica albuginea or testis. It had a firm consistency and on section presented a fairly homogeneous white surface. Microscopical examination revealed a pleomorphic sarcoma composed of sheets of spindle cells with elongated nuclei and bipolar cytoplasmic prolongations and areas of pleomorphic cells with poorly defined cell outlines containing variable amounts of eosinophilic cytoplasm and prominent nuclei. Occasional multinucleated giant cells were noted and large cells with abundant eosinophilic cytoplasm and irregular outlines (Fig. 1). Areas of necrosis were widespread and in other regions the tissue had a loose myxomatous appearance. Numerous thin-walled vessels permeated the tumour and a few fibrous septa divided it into ill-defined lobules. The diagnosis of rhabdomyosarcoma was confirmed by finding cross striations (Fig. 2).

Thirteen days post-operatively a swelling, approximately thumb-tip size was noted in the scrotum, adherent to the scrotal skin. This was thought to be a haematoma, but the possibility of a recurrence was also considered. He was discharged from hospital on the 15th post-operative day and readmitted 2 months later with increasing pain in the scrotum and also in the right buttock and thigh, associated with numbness. He also had a cough and had lost 2 stone weight. Enlarged inguinal lymph glands were palpable and a mass of para-aortic glands was felt above the umbilicus. X-ray chest revealed numerous secondaries and collapse was noted in L4. He was treated chiefly with sedation and largactil. His progress was rapidly downhill with increasing pain, urinary incontinence, stridor and forced expiration, vomiting and abdominal distension. Terminally he developed numbness of the left foot, gross oedema of the penis and scrotum and a swelling of the parotid region.

*Case* 2, *D.D.P. aged* 18 *years*.—This patient felt well until 2 months prior to admission when he noticed a slight swelling in the base of the right testis. The swelling was never painful or tender but gradually increased in size, spreading to involve the whole testis and progressing upwards into the external inguinal ring. This was 3 weeks prior to admission and soon afterwards he developed pain in his right iliac fossa. The increasing pain and swelling led him eventually to consult his G.P., who referred him to hospital. No history of trauma was given.

Systematic questions revealed no abnormality and he had no significant past or family history. B.P. 115/70. The only positive findings were confined to the scrotum and abdomen. A large swelling approximately $15 \times 6$ cm. was obvious in the right side of the scrotum, extending up through the superficial inguinal ring for about 6 cm. The mass was very hard, with a smooth surface.

No fluctuation, transillumination or tenderness was elicited. The left testis felt normal. Tenderness was observed in the right iliac fossa and right renal area. No hepato- or splenomegaly was found and the kidneys were not palpable. A diagnosis of testicular neoplasm was made. No opacity was found on chest X-ray. Operation was performed with removal of the right testis and cord up to the internal ring. On section a little (2–3 ml.) slightly blood-stained fluid escaped from the tunica vaginalis. The specimen was sent for pathological examination (Fig. 3). It consisted of testis, epididymis and cord weighing 425 g. and contained a large circumscribed tumour 15 × 10 cm. The tumour arose in the epididymis or lower part of the spermatic cord and not in the testis, though the tunica albuginea appeared partly infiltrated. The tumour was firm, but not hard, and a pattern of interweaving bundles could be seen on its cut surface. The tissue was pale and fleshy with occasional areas of necrosis. Histologically the tumour showed considerable variation, being mostly composed or irregular polygonal cells but with many groups of pleomorphic cells, some containing abundant eosinophilic cytoplasm. A few strap cells were present (Fig. 4) and cross striations could be identified. The upper part of the cord was free from tumour.

The patient was discharged 10 days post-operatively but required readmission 1 month later when one of his legs became swollen. Later both legs were affected and he complained of pain in his back and extreme weight loss. His behaviour became peculiar, varying from agitation to depression. The first X-ray evidence of metastases appeared 2 months post-operatively when increased shadowing was observed in the left lower lobe. Progression from this point was rapidly downhill with the development of a large mass in the epigastrium and chest pain of increasing severity. Terminally, haemoptyses occurred and the patient died 5 months after operation and 7 months from the first evidence of testicular enlargement.

Post-mortem examination revealed dilated subcutaneous veins over the abdomen. Serosanguinous effusions were noted in both pleural cavities and the peritoneal cavity. A large tumour mass, approximately 2 kg., was present in the posterior peritoneum, extending into the mesentery. The tumour was based on the para-aortic lymph nodes, but had grown far beyond them, separating the aorta and inferior vena cava, and compressing and infiltrating the latter, with resulting partial occlusion by organised thrombus. The tumour mass also extended into the right iliac fossa and upwards around the right kidney and ureter. The liver contained only a few very small tumour nodules in the right lobe, in comparison with the lungs which were extensively studded with soft pale tumour nodules, measuring up to 5 cm, in diameter, (Fig. 5). The tumour was mainly peripherally placed and the pleura extensively involved. The mediastinal lymph nodes contained secondary tumour. Microscopical examination revealed a similar but more variable appearance than in the biopsy specimen, with foci of round cells containing heavily stained but essentially vesicular nuclei and little cytoplasm. Well differentiated muscle fibres were infrequent although the secondaries in the lungs contained many strap cells and muscle fibres (Fig. 6). In this situation and also in the liver, there was a remarkable degree of preservation of the original tissue outlines and many islands of liver cells remained among the tumour cells. No tumour was found in the obstructed inferior vena cava. Tumour infiltrated lymph nodes to varying degrees.

*Case* 3, *J.M. aged* 72 *years.*—Three years prior to admission this elderly man noticed a small lump in his right testis following an injury and consulted his family doctor. He was advised against operation and given a truss to wear. He had an occasional twinge of non-radiating pain in his right testis. One year before admission, he had a groin injury, following which he noticed a progressively enlarging suprapubic mass. No history of frequency or dysuria was obtained. On examination the suprapubic mass felt roughly circular, mobile (in a vertical plane, but not in a horizontal plane along the inguinal ligament) firm to hard in consistency and the skin moved over it. The right testicle felt irregular. No enlarged inguinal glands were found and the kidneys were not palpable. A diagnosis of right testicular seminoma was made and operation carried out 1 week after admission. The right half of the scrotum and right testis were excised and the tumour itself mobilized and removed by excising part of the external and internal oblique and transversus muscle. Tumour was found adherent to the peritoneum which was opened and the iliac arteries and aorta palpated, but no enlarged lymph nodes were found.

The specimen received for pathological examination consisted of testis, surrounding tissues, spermatic cord and tumour. The tumour measuring 9 × 12 × 6 cm. arose in the region of the cord and was generally white with areas of necrosis. Histologically it was composed predominantly of bundles of interwoven spindle cells with a few bands of dense collagenous fibrous tissue dividing it into lobules. Occasional myxomatous areas were noted. However, marked cellular pleomorphism was present in areas and many cells exhibited abundant eosinophilic cytoplasm, varying from tapering processes to large cytoplasmic masses with peripheral nuclei (Fig. 7). Vacuolation was fairly common, some cells having a " spider " appearance. Binucleated and multinucleated cells were encountered frequently and mitoses were occasionally seen. Strap cells and racquet cells were also present and the finding of a few cells with cross-striations led to a diagnosis of rhabdomyosarcoma (Fig. 8).

As it was felt that this was not radiosensitive the patient was discharged when he had recovered from his operation, to convalesce at home. His condition deteriorated rapidly, irregular liver enlargement was noted and he died 1 month later. Autopsy was not performed.

*Case* 4, *J.S. aged* 72.—Approximately 1 month before admission, this man noticed a swelling in his left groin, associated with pain on straining. Otherwise his general health was good and there were no urinary symptoms. On examination a hard, painful swelling was found by the G.P. and the patient was sent to hospital for investigation. On examination 2 masses were felt, 1 overlying and fixed to the left os pubis and a second discrete hard nodule in the adjacent cord. A tentative diagnosis of osteitis pubis was made, or possibly a tumour arising from Paget's disease of bone. He was admitted to hospital and X-ray showed no bony attachment. A laparotomy was carried out on 5.4.1965 via a left inguinal incision and the mass overlying the left os pubis dissected out fairly easily and was found to be a 5 × 4 cm. well circumscribed solid tumour arising within the cord. A second nodule was situated just above the upper pole of the epididymis. The testis itself presented no particular abnormality on gross inspection. The cord was cleared and divided above the deep inguinal ring, thus removing the testis and tumours. The specimen was sent for pathological examination where the lack of pathology in the testis was confirmed. A white somewhat irregular shaped tumour nodule 4·5 cm. in diameter was situated in relation to the vas. This had a whorled appearance on section. The second nodule, about 1 cm. in diameter, was situated just above the upper pole of the epididymis. Histologically both tumours were similar (Fig. 9) and showed spindle-shaped cells interlaced with some collagen bundles. Vascular spaces were fairly prominent. Little cellular pleomorphism was observed and there were few mitoses. Occasional multinucleated muscle cells with abundant pink cytoplasm were found. The presence of 2 nodules, the pleomorphism on histological examination and the failure of the smaller nodule to appear encapsulated suggested that they should be designated as leiomyosarcoma rather than leiomyoma.

Post-operative recovery was rapid and uncomplicated and the patient was discharged on 25.4.1965. Follow-up revealed no recurrence of the tumour until 13.3.1967 when a firm swelling was noted in the left groin. He was readmitted and the lesion removed. An 85 g. nodule of firm fibro-fatty tissue and tumour was received for pathological examination. No lesion quite so distinct as the previous tumour was found. Scattered throughout dense collagenous fibrous tissue were spindle cells and occasional cells with large hyperchromatic nuclei, suggesting a tumour recurrence. Very few mitotic figures were present. Apart from the occasional bizarre cells, the lesion suggested a keloid scar formation, but a few cells could not be considered as reactive. However it was felt that the tumour was of low grade malignancy and whilst it might recur, it would be unlikely to disseminate. Post-operative recovery was uneventful and follow-up has revealed no recurrence or dissemination to date.

*Case 5, H.S. aged 60 years.*—This man presented with a swelling in his scrotum of 2 years duration. No history of trauma was obtained and there was no pain or tenderness. On examination a tense cystic swelling was noted above the left testis, and a diagnosis of cyst of the cord was made. No other physical abnormality was detected and operation was performed 2 months later with removal of testis, spermatic cord and tumour. The testis and tumour mass received for pathological examination weighed 175 g. The tumour had a greatest diameter of 5 cm. and was situated in relation to the spermatic cord, well clear of the testis and body of the epididymis. On section it was whitish in colour with some areas of degeneration. Histological examination of testis and epididymis showed only atrophy of testicular elements. Tumour was seen involving the substance of the cord suggesting an origin therein. It was composed of extremely pleomorphic cells, often with abundant pink cytoplasm and vesicular nuclei (Fig. 10). Many cells were multinucleated and in some a large irregular single nucleus occupied the major portion of the cell. Peripheral vacuolation of the cytoplasm of some cells gave them the appearance of spider cells. Only rarely were cells spindle-shaped or arranged in bundles. Special stains did not reveal any myofibrils or cross-striations, though granules in many cells suggested the possibility of immature striations. It was considered best to regard the tumour as a pleomorphic or undifferentiated sarcoma. It was thought that radiotherapy would be of little value and following an uneventful recovery he was discharged one week post-operatively.

Four months later a recurrence was noted at the site of previous operation and on removal was found to be histologically very similar to the previous biopsy. From this stage the patient's condition deteriorated rapidly with enlargement of the liver which felt nodular and with symptoms and signs of lung involvement. He died within 2 months of the recurrence, from generalised metastasis. Necropsy was not performed.

*Case* 6, *J.H. aged* 50 *years.*—In November, 1959 this patient presented with prolapsing haemorrhoids and was boarded for haemorrhoidectomy. However, before this operation was carried out he presented again in August, 1960 with a swelling in his left groin of approximately 6 weeks duration, thought to be an irreducible inguinal hernia. He volunteered that the swelling was smaller in the morning than the evening and that he developed slight pain in this region after standing for a while. He felt that he might have strained himself about 6 weeks previously when he started getting slight pain, e.g., after stretching. A left irreducible inguinal hernia was found on examination but it was felt that a firm mass was also situated in the region of the cord. This did not transilluminate, was not attached to the hernia, but appeared to be attached to the testis. He was admitted directly and operation carried out through a left inguinal incision. The lump was mobilised from the scrotum and separated from the cord. A narrow fatty pedicle passed into the retroperitoneal tissues via the deep inguinal ring. A small hydrocele was turned inside out, the deep ring tightened and the wound closed. The specimen received for pathological examination weighed 145 g. and consisted of fat and predominantly fibrous tissue of a rather myxomatous character (Fig. 11). The latter feature was considered disturbing and since some markedly irregular cells were seen, it was thought that the tumour would be likely to recur if not completely removed and it was uncertain from the specimen itself whether or not this was so. The tumour was classed as a myxofibroma with a possible tendency to local malignancy but unlikely to metastasise. Recovery was smooth and he was followed-up at 3-monthly intervals. Two months later he had a haemorrhoidectomy. In December, 1963 he was admitted with a 17-hour history of crampy abdominal pain, nausea and vomiting. Slight tenderness was found in the right iliac fossa but nothing else. He recovered rapidly and was discharged 4 days after admission. He continued to attend the follow-up clinic and in March, 1964, was found to have a positive Wasserman reaction and a positive Kahn test. Reiter's protein C.F.T. was positive and he had a mild anaemia. In May, 1964 a palpable right kidney was detected and an I.V.P. showed a possible calculus at the lower end of the right ureter, but good excretion in both kidneys. A cystoscopy, with right retrograde pyelography was arranged but 2 weeks later he was readmitted for excision of a recurrence of the swelling in his left groin. Operation was carried out and on this occasion the testis and spermatic cord were removed with the swelling and sent for pathological examination. The 115 g. specimen contained several tumour nodules measuring up to 3 cm. in diameter, situated along the spermatic cord. The testis and epididymis appeared normal. The tumours were composed of fairly mature fibrous tissue, in which a large number of fat-filled cells were detected. Often these cells contained several globules of fat and frequently the nuclei were bizarre and multiple. The presence of these abnormal fat cells indicated a diagnosis of fibroliposarcoma, though it was still considered that the tumour was more likely to be locally malignant only and distinct metastasis unlikely. Post-operatively he recovered rapidly, complaining only of pain in the distribution of the ilio-inguinal nerve and phantom testicular pain. He has been followed-up since his discharge and remains free from recurrence.

*Case* 7, *K.B. aged* 4 *years.*—A rhabdomyosarcoma of the tunica vaginalis testis was seen recently in a 4-year-old boy. Following orchidectomy it was noted that the tumour did not involve the testis itself (Fig. 12) and histological examination revealed abundant cross striations. In consultation with the surgeon and radiotherapist it was decided to carry out lymphangiography before further consideration of irradiation or retroperitoneal node dissection. The results will be reported later.

*Case* 8, *J.M. aged* 51 *years.*—A full clinical history and follow-up were not available. The patient presented with a large scrotal swelling, which was excised and sent for histological examination. The specimen consisted of testis, epididymis and tumour (Fig. 13). A large 9·5 × 6·5 × 5 cm. tumour showing extensive central necrosis displaced the testis and epididymis, which were not involved. The tumour was partially surrounded by a fibrous capsule and contained several blood vessels. Histological examination revealed dense interweaving bands of eosinophilic spindle cells and myxomatous areas with occasional multinucleated cells. There was marked necrosis in areas and special stains revealed longitudinal myofibrils. A diagnosis of leiomyosarcoma was made and neither radiotherapy nor drug therapy was considered of value. The patient's course was rapidly downhill with death occurring a few months later.

*Case* 9, *A.G. aged* 57 *years.*—Five years prior to admission this man fell off his bicycle and damaged his scrotum. Three years later he noticed a small lump in the right side of his scrotum, which began to enlarge 7 weeks before admission. The only relevant findings were in his scrotum and at operation testis and cord were removed. A small hard whitish tumour mass was noted at the lower pole of the epididymis and this was sent to the Pathology Department. Histological examination showed this tumour to consist of spindle cells arranged in small interlacing bundles or whorls. Pleomorphic cells were scattered throughout, though the general appearance was of a leiomyosarcoma of low grade malignancy.

---

### EXPLANATION OF PLATES

FIG. 1.—Note the pleomorphic nature of the tumour, occasional giant forms having multiple nuclei and abundant eosinophilic cytoplasm. H. & E. ×350.

FIG. 2.—Cross-striations are present in well-formed muscle fibres and in immature cells. Heidenhain ×1200.

FIG. 3.—A large solid tumour involves the epididymis and spermatic cord. The tunica albuginea is partly infiltrated. ×2/3.

FIG. 4.—Note the variation in pattern with myxomatous and highly compact areas adjacent to one another. Strap cells and spindle cells are present. H. & E. ×250.

FIG. 5.—Secondary deposits of rhabdomyosarcoma are situated in the pleura and throughout the lung tissue. ×½.

FIG. 6.—" Strap " cells and muscle fibres are prominent in lung tissue surrounding bronchioles. H. & E. ×140.

FIG. 7.—Abundant eosinophilic cytoplasm and bizarre nuclei are seen in giant cell forms with occasional cytoplasmic vacuolation. H. & E. ×300.

FIG. 8.—A typical " tadpole " or " racquet " cell lies adjacent to a cell showing cross-striations. Beading of the cytoplasm of some cells may represent immature striations. Heidenhain ×1300.

FIG. 9.—Interweaving bundles of smooth muscle cells showing some nuclear pleomorphism and prominent nucleoli. H. & E. ×510.

FIG. 10.—The undifferentiated nature of this highly cellular tumour is shown. Mitoses and nuclear pleomorphism are prominent. H. & E. ×280.

FIG. 11.—Occasional bizarre nuclei are seen in rather myxomatous fibrous tissue. H. & E. ×210.

FIG. 12.—This rhabdomyosarcoma involves the tunica vaginalis only, the testis, epididymis and cord being free of tumour. ×1.

FIG. 13.—Leiomyosarcoma involving the spermatic cord and displacing the testis and epididymis. Necrosis is extensive. ×¾.

1

2

3

4

5

6

7

Alexander.

8

9

10

Alexander.

11

12

13

Alexander.

Special stains revealed longitudinal myofibrils and confirmed the diagnosis. Follow-up is not available.

*Case* 10, *F.H. aged* 51 *years*.—This man gave a history of traumatic injury to his right testis 27 years previously. A hard irregular swelling in this region had gradually increased in size and had become tender over the 6 months before admission. He had a past history of rheumatic heart disease and a mitral systolic murmur was noted on examination. The only other finding was a hard irregular swelling about 4 cm. in diameter at the upper pole of the testis. This was removed and found to lie in relation to the epididymis, but not involving it. The tumour appeared to be of smooth muscle origin with some areas of degeneration and haemorrhage. There was no invasion of surrounding tissue but the mild pleomorphism observed gave considerable concern. It was thought that the tumour was unlikely to metastasise, but might well recur. The patient was discharged and recurrence with dissemination occurred.

*Cases* 11 *and* 12.—The clinical details of these cases are not available but macroscopic specimens and histological sections have been studied.

*Case* 11.—This 9 × 6 × 6 cm. tumour arose in relation to the spermatic cord and did not involve the testis. Histological examination revealed a highly cellular tumour consisting of bands of spindle cells showing considerable pleomorphism. The lack of eosinophilic cytoplasm and myofibrils and the staining reactions suggested a diagnosis of fibrosarcoma.

*Case* 12.—This 5 cm. in diameter firm white tumour with a whorled appearance consisted of sheets of round, oval and spindle cells with areas of necrosis. The tumour infiltrated around fat and blood vessels in relation to the vas. No testicular involvement was seen. No myofibrils or cross-striations were identified and a diagnosis of undifferentiated sarcoma was made.

### DISCUSSION

*Rhabdomyosarcoma*.—As previously indicated paratesticular rhabdomyosarcomas are very rare, Dixon and Moore (1952) encountering only one in 2,000 tumours of the male genital system. Approximately 50 well-authenticated cases have now appeared in the literature, but unfortunately these reports rarely include adequate documentation of follow-up and only 3 post-mortem reports are available. It therefore seemed worthwhile to add 4 cases, 3 with follow-up until death and 1 with necropsy.

It has generally been accepted that sarcomas tend to recur locally or spread by the blood stream, following invasion of the thin sinusoidal channels within them. However, as more of these cases are reported there is an increasing awareness of lymphatic spread, at least as commonly as blood spread. Many reports refer to, retroperitoneal masses (Ravich, Lerman and Sands, 1964; Areán and Kreager 1965). Limited post-mortem findings as reported by the latter indicated retroperitoneal metastases, invasion of the pancreas and extension of the porta hepatis without liver involvement and no clinical evidence of lung secondaries. Such findings suggest lymphatic spread without vascular spread. Considering the prognosis of their 11 cases, Gowing and Morgan did not comment on the mode of spread apart from stating that one case had massive intra-abdominal lymph-node involvement within 1 month of removal of the primary growth. It must not be concluded from the foregoing statements that lymphatic spread is necessarily the more common method of spread, since one may also find reported clinical findings, e.g. infiltrated sternal and iliac marrow in the absence of palpable lymph nodes strongly suggesting vascular dissemination, (Areán and Kreager, Case 1). Severe cough and right chest pain, with X-ray evidence of massive metastases were the first evidence of tumour spread as reported by Hoffman and Baird (1960), again suggesting primary vascular spread, though this may of course have followed lymphatic involvement to thoracic duct level. In Case 1 described above little conclusive evidence can be drawn as to the relative importance of lymphatic and blood spread, since enlarged lymph nodes were palpable and X-ray evidence of metastases was noted in the lungs and vertebrae when the patient was readmitted 2 months post-operatively. In Case 2 however, there would appear to be good reason to suspect lymphatic involvement first, since his symptoms on readmission 1 month post-operatively were of lymphatic obstruction with swelling of one of his legs. The first evidence of chest involvement was radiological, 1month later. At necropsy evidence of direct, lymphatic and blood spread were noted, the massive retroperitoneal tumour appearing to be based on the para-aortic nodes, with histological evidence of lymph node involvement. Areán and Kreager have suggested that lymphangiograms should prove useful in evaluating the extent of spread of such tumours, but no report of such investigation has been found. In the absence of distant metastases at the time of surgery, retroperitoneal lymph node dissection has been advocated by Gray and Biorn (1955). Since metastases often occur within a short time of surgery, it would seem possible that dissemination may have occurred already or may be initiated by handling the tumour, e.g. Case 3, as reported, had a 3 year history of scrotal swelling pre-operatively, yet died within 1 month of surgery. It would therefore appear worthwhile to carry out removal of local lymph nodes, with a minimum of handling of the testis, epididymis and vas, prior to clamping the cord above such tumours, especially if lymphangiograms show evidence of lymph node involvement.

From a review of the literature it would seem impossible at present to offer an accurate prognosis in these cases. Not until this decade has there been a reported survival for more than 2 years. A remarkable case was then described by Walker and Cameron when a 16-year old with rhabdomyosarcoma of the cord survived 7 years despite the histological finding of dense masses of highly anaplastic tumour tissue in many of the lymphatics and some of the veins at the time of removal. Irvine *et al.* (1962) described a $3\frac{1}{2}$-year-old surviving 5 years after surgical excision, and this was followed by 3 cases reported by Holtz and Abell (1963) surviving 2, 3 and 12 years post-operatively.

Paratesticular rhabdomyosarcomas are typically tumours of the younger age groups being only rarely reported after the second decade as shown in Fig. 14.

The present report contains a case in the eighth decade. Only one of these elderly patients was known to be alive 1 year after tumour excision. From an analysis of the 30 cases with accurately reported survival, it would appear that prognosis bears a limited relationship to age (Table I). The average age of cases reported to be well 2 years or more after surgery is $5\frac{3}{4}$ years, only the 16-year-old of Walker and Cameron being more than 7 years old at orchidectomy. In comparison the average age of those dying within 2 years of surgery is 23 years. Of the 9 patients in the first decade at the time of detection, only 3 have died, whereas in all other decades only 8 patients have survived more than 1 year and only one is known to have survived more than 2 years.

FIG. 14.—Age incidence of paratesticular rhabdomyosarcoma.

TABLE I.—*Relationship of Age at Operation to Survival*

| Age | Survival | Age | Survival | Age | Survival |
|---|---|---|---|---|---|
| 3 months | 12 years | 10 years | 13 months | 17 years | 4 months |
| 21 months | 7 months | 10 years | $1\frac{1}{2}$ years | 17 years | 1 year* |
| 3 years | 3 years* | 14 years | 16 months | 18 years | 5 months |
| $3\frac{1}{2}$ years | 5 years* | 14 years | 9 months | 19 years | 1 year |
| 4 years | —* | 15 years | 5 months | 19 years | 2 years |
| 4 years | 16 months | 16 years | 7 years* | 20 years | 6 months |
| $4\frac{1}{2}$ years | 7 years* | 16 years | 9 months | 62 years | post-op |
| 5 years | $3\frac{1}{2}$ months* | 16 years | 3 months | 72 years | 1 month |
| 6 years | 10 months* | 16 years | $1\frac{1}{2}$ years | 75 years | 1 year* |
| 7 years | 2 years* | 17 years | $3\frac{1}{2}$ years | 79 years | 9 months |

* Alive at time of reporting.

The improved survival in the young may be related to earlier medical consultation following detection of the scrotal swelling by a parent, whereas the mass may be the only finding in the adult who otherwise feels so well that he does not consult his physician for several months or even years. No definite correlation exists however between duration of symptoms and post-operative survival, though it is worth noting that all but one of the patients still alive 2–12 years post-operatively were operated on within 2 months of tumour detection. Nine other patients with symptoms for 2 months or less died within $1\frac{1}{2}$ years of operation.

As reported in the literature, treatment other than operative removal provides little benefit. Radiotherapy is of very doubtful benefit and only Hoffman and Baird report a response to chemotherapy. Following massive doses of Amethopterin these writers reported 2 remissions of 6 weeks and 4 weeks with X-ray evidence of regression of pulmonary metastases and marked clinical improvement, but following these remissions deterioration was rapid with death 9 months after orchidectomy.

The site of origin of paratesticular rhabdomyosarcomas is variously reported as cremasteric muscle, embryonic rests of totipotent cells, metaplasia from the muscle of the vas deferens, or in the tunica vaginalis.

The typical histological appearance of rhabdomyosarcoma has been outlined by Stout (1946) and Horn and Enterline (1958), and does not require further consideration here. Suffice it to say that the tumours described fulfilled the required criteria and cross-striations were demonstrated.

*Leiomyosarcoma.*—An excellent recent review of the 22 previously reported cases of leiomyosarcoma, the rarest sarcoma of the spermatic cord, has been presented by Kyle. He pointed out that they were considered to have developed in previously benign lesions in 5 cases and that the probable site of origin was the blood vessels in the cord. In contradistinction to rhabdomyosarcoma, the age incidence ranged from 15 years to 78 years, only 1 of the patients being under 20 years. The 23rd case of leiomyosarcoma reported here remains well 3 years after primary removal and 1 year after resection of a recurrence. Prognosis based on our present knowledge is extremely variable, death occurring as early as 4 months after removal (Meinardi, 1961) or survival continuing 22 years post-operatively (Thompson, 1936). The significance, if any, of radiation and lymph node dissection will only be known when many more cases with satisfactory follow-up are described.

SUMMARY

Twelve cases of paratesticular sarcoma occurring over the past 35 years in Northern Ireland, population $1\frac{1}{2}$-million, are presented. Six of these are described fully with follow-up—3 rhabdomyosarcomas with 1 autopsy, a leiomyosarcoma, an undifferentiated sarcoma and a fibroliposarcoma. Six other cases are described briefly. Rhabdomyosarcomas are discussed in detail and the relevant literature reviewed. Lymphangiography is suggested as an investigation which should be useful in deciding therapy and the prognosis in the young appears to be much better than has been generally believed.

Acknowledgment is made to Professor Sir John Biggart, C.B.E., Professor E. F. McKeown and Dr. J. E. Morrison for helpful criticism and advice; all those surgeons who have allowed their cases to be presented and Mr. Mehaffey for his photography.

### REFERENCES

Areán, V. M. and Kreager, J. A.—(1965) *Am. J. clin. Path.*, **43**, 418.

Dixon, F. J. and Moore, R. A.—(1952), In ‘Tumours of the Male Sex Organs’, A.F.I.P. Atlas of Tumor Pathology, Sec. VIII, Page 138.

Fox, J. A. and Collier, R. L.—(1967) *Am. Surg.*, **33**, 483.

Gowing, N. F. C. and Morgan, A. D.—(1964) *Br. J. Urol.*, **36** (Suppl. 2) 78.

GRAY, C. P. AND BIORN, C. L.—(1955) *J. Urol.*, **74**, 402.
HOFFMAN, W. W. AND BAIRD, S. S.—(1960) *J. Urol.* **84**, 376.
HOLTZ, F. AND ABELL, M. R.—(1963) *Cancer, N.Y.*, **16**, 982.
HORN, R. C. JR., AND ENTERLINE, H. T.—(1958) *Cancer, N.Y.*, **11**, 181.
IRVINE, E. W., BERG., O. C. AND NELSON, R.—(1962) *New Engl. J. Med.*, **266**, 994.
KYLE, V. N.—(1966) *J. Urol.*, **96**, 795.
MEINARDI, E.—(1961) *Minerva chir.*, **16**, 1109.
RAVICH, L., LERMAN, P. H. AND SANDS, A.—(1964) *J. Urol.*, **92**, 144.
STOUT, A. P.—(1946) *Ann. Surg.*, **123**, 447.
THOMPSON, G. J.—(1936) *Surgery Gynec. Obstet.*, **62**, 712.
WALKER, W. F., AND CAMERON, H. MCD.—(1961) *Br. J. Surg.*, **49**, 319.